# Supplementary material for: Methodological considerations for assessing elder mistreatment of older adults with cognitive impairment: A scoping review protocol
Source: PLoS One. 2025 Mar 27;20(3):e0320689. doi: 10.1371/journal.pone.0320689 (PMC11949334; doi:10.1371/journal.pone.0320689)
Supplement: S1 Fig — (DOCX) [file pone.0320689.s001.docx]

**Identification of studies via other methods**

**Identification of studies via databases and registers**

Records identified from:

Websites (n =?)

Organisations (n =?)

Citation searching (n =?)

etc.

Records removed *before screening*:

Duplicate records removed

(n = 2270)

Records marked as ineligible by automation tools (n = 0)

Records removed for other reasons (n = 0)

Records identified from*:

Databases (n = 4)

Registers (n = 0)

**Identification**

Records screened

(n =?)

Records excluded**

(n =?)

Reports not retrieved

(n = ?)

Reports sought for retrieval

(n =?)

Reports sought for retrieval

(n =?)

Reports not retrieved

(n =?)

**Screening**

Reports assessed for eligibility

(n =?)

Reports excluded:

Reason 1 (n =?)

Reason 2 (n =?)

Reason 3 (n =?)

etc.

Reports assessed for eligibility

(n =?)

Reports excluded:

Reason 1 (n =?)

Reason 2 (n =?)

Reason 3 (n =?)

etc.

Studies included in review

(n =?)

Reports of included studies

(n =?)

**Included**

This work is licensed under CC BY 4.0. To view a copy of this license, visit <https://creativecommons.org/licenses/by/4.0/>

Reference

1. Page MJ, McKenzie JE, Bossuyt PM, Boutron I, Hoffmann TC, Mulrow CD, et al. The PRISMA 2020 statement: An updated guideline for reporting systematic reviews. BMJ. 2021;372: n71. doi: 10.1136/bmj.n71.
